# Scale-up of home-based management of malaria based on rapid diagnostic tests and artemisinin-based combination therapy in a resource-poor country: results in Senegal

**DOI:** 10.1186/1475-2875-11-334

**Published:** 2012-09-25

**Authors:** Sylla Thiam, Julie Thwing, Ibrahima Diallo, Fatou B Fall, Mame B Diouf, Robert Perry, Medoune Ndiop, Mamadou L Diouf, Moustapha M Cisse, Mamadou M Diaw, Moussa Thior

**Affiliations:** 1 African Medical and Research Foundation, Nairobi, Kenya; 2 Centers for Disease Control and Prevention, Atlanta, GA, USA; 3 Programme National de Lutte contre le Paludisme, Dakar, Senegal; 4 Centre d’Orientation Scolaire et Professionnelle de Diourbel, Diourbel, Senegal

**Keywords:** Malaria, Treatment, Diagnosis, Community, Home-based management

## Abstract

**Background:**

Effective case management of malaria requires prompt diagnosis and treatment within 24 hours. Home-based management of malaria (HMM) improves access to treatment for populations with limited access to health facilities. In Senegal, an HMM pilot study in 2008 demonstrated the feasibility of integrated use of RDTs and ACT in remote villages by volunteer Home Care Providers (HCP). Scale-up of the strategy began in 2009, reaching 408 villages in 2009 and 861 villages in 2010. This paper reports the results of the scale-up in the targeted communities and the impact of the strategy on malaria in the formal health sector.

**Methods:**

Data reported by the HCPs were used to assess their performance in 2009 and 2010, while routine malaria morbidity and mortality data were used to assess the impact of the HMM programme. Two high transmission regions where HMM was not implemented until 2010 were used as a comparison.

**Results and discussion:**

From July 2009 through May 2010, 12582 suspected cases were managed by HCPs, 93% (11672) of whom were tested with an RDT. Among those tested, 37% (4270) had a positive RDT, 97% (4126) of whom were reported treated and cured. Home care providers referred 6871 patients to health posts for management: 6486 with a negative RDT, 119 infants < 2 months, 105 pregnant women, and 161 severe cases. There were no deaths among these patients. In 2009 compared to 2008, incidence of suspected and confirmed malaria cases, all hospitalizations and malaria-related hospitalizations decreased in both intervention and comparison regions. Incidence of in-hospital deaths due to malaria decreased by 62.5% (95% CI 43.8-81.2) in the intervention regions, while the decrease in comparison regions was smaller and not statistically significant.

**Conclusion:**

Home-based management of malaria including diagnosis with RDT and treatment based on test results is a promising strategy to improve the access of remote populations to prompt and effective management of uncomplicated malaria and to decrease mortality due to malaria. When scaled-up to serve remote village communities in the regions of Senegal with the highest malaria prevalence, home care providers demonstrated excellent adherence to guidelines, potentially contributing to a decrease in hospital deaths attributed to malaria.

## Background

Effective management of malaria cases requires prompt diagnosis and effective treatment within 24 hours. Despite the efforts made by countries to scale up artemisinin-based combination therapy (ACT) and rapid diagnostic tests (RDTs) in health facilities, most people who are vulnerable to malaria live far from health facilities and have limited access to diagnosis and treatment. The lack of access to malaria services, particularly in remote areas, is a barrier to early and appropriate malaria case management, thereby leading to more complicated disease and malaria-related deaths. More than 90% of sick children in rural areas in Senegal receive their first treatment at home. Unfortunately, most of the time, they receive inappropriate self-medication [1].

Expanding malaria diagnosis and treatment is critical to achieving universal access to case management measures and to eliminating deaths due to malaria by 2015. The World Health Organization (WHO), therefore, recommends home-based management of malaria (HMM) as a means to increase access to prompt and effective treatment of malaria [2]. Bringing treatment closer to home through the implementation of HMM should improve access to malaria case management services in areas with scarce health facilities and qualified human resources, as is typically found in rural Africa [3-5]*.* A number of studies, some of them conducted under the leadership of the WHO, have demonstrated its feasibility and efficacy [6-9], and several studies have evaluated the introduction of ACT through HMM programmes [10,11].

The introduction of malaria case management with parasitological diagnosis adds a layer of complexity, with questions as to the ability of community level volunteers to adequately perform the RDTs and administer treatment according to test results. Recently, successful introduction of RDTs into HMM programmes has been reported in several African countries [12-17]. However, most of these studies are pilot experiences implemented in limited areas and targeted only children under five. Thus the interventions did not reach all vulnerable populations, particularly within the context of universal access to diagnosis and treatment, and did not maximize public health impact.

In Senegal, case management of malaria in public health facilities was scaled up nationwide with ACT in 2007 and RDTs in 2008 [18]. However, despite scaling up ACT and RDTs in all public health facilities, challenges still existed relating to early and adequate care of malaria cases in Senegal, particularly in rural areas with limited access to health services. In 2008, an HMM pilot study involving 20 villages located more than 5 km from the nearest health facility, each with a volunteer home care provider (HCP) trained to manage uncomplicated malaria using RDTs and ACT, demonstrated the feasibility of integrated use of RDTs and ACT in the community [14]. Based on encouraging results from the pilot phase, the Ministry of Health decided to scale up HMM, reaching in total 408 remote villages in 2009 and 861 remote villages in 2010 [19].

Data reported by HCPs and district level reporting of malaria morbidity and mortality were used to evaluate the impact of the scale-up of home-based management of malaria using RDTs and ACT to provide case management based on parasitological diagnosis of malaria.

## Methods

### Setting

**Figure 1:**
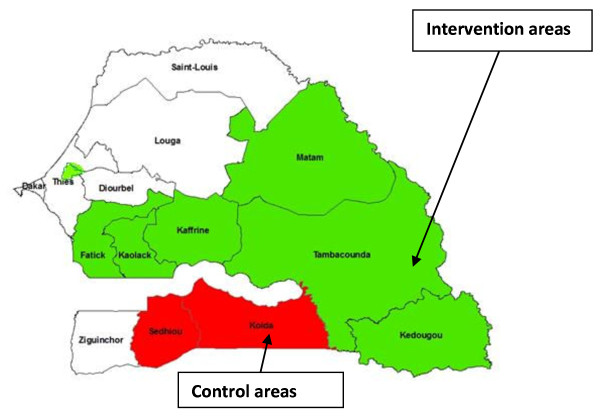
Map of regions in which home-based management of malaria was implemented, and the two comparison regions.

Senegal is a West African country where malaria is endemic, with a high transmission period during the rainy season from July to November. The entire population is at risk and *Plasmodium falciparum* accounts for 100% of reported malaria cases [20]*.* In 2009, HMM was scaled-up in 25 rural districts located in seven high prevalence regions in southern and eastern Senegal with high rates of malaria transmission and malaria-related morality (Figure 1). The total population of the regions in which HMM was introduced was 3,202,760 inhabitants, though the population served by the HMM programme was much smaller, with a typical village size averaging 500 inhabitants. Scale up of the HMM strategy commenced in January 2009, with selection of HCPs in 408 villages either located ≥5 km from the nearest health facility or with difficulties accessing the closest health facility, either throughout the year or during the rainy season. Data recording began in July 2009, at the beginning of malaria transmission season. During 2010, the additional 453 villages were added prior to the malaria transmission season, bringing the total number of villages to 861, the number of districts involved to 32, and adding two additional high prevalence regions.

### Intervention

#### Training

**Figure 2:**
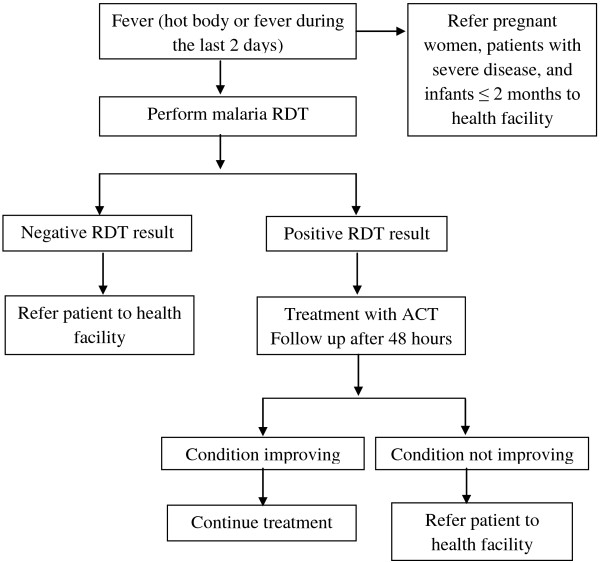
Algorithm for case management of fever by HCPs.

In each village a volunteer HCP able to read and write was chosen by the community. They were trained on (i) malaria case management using RDTs and artesunate-amodiaquine, and (ii) referral of RDT negative patients, those with severe disease, and all malaria cases in pregnant women and children less than two months and (iii) collecting data using standardized tools. Job aids with a clear algorithm were developed by the NMCP and used by the HCPs to manage patients (Figure 2).

**Figure 3:**
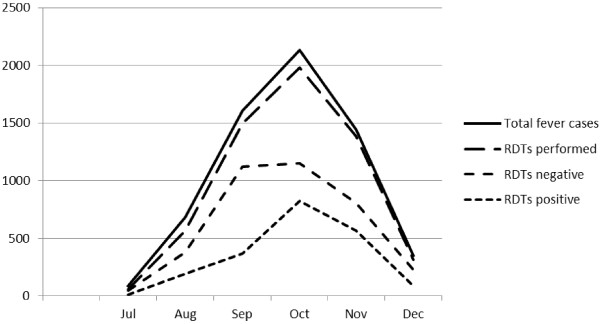
Total numbers of cases seen monthly by home care providers, rapid diagnostic tests performed, and test results: July through December 2009.

A cascade model was adopted to conduct trainings. First, in each region all District Health Teams (DHTs) and the regional health team received two days of training as trainers by the National Malaria Control Programme (NMCP) personnel. The DHTs, supported by the regional team and under the supervision of the NMCP team, trained HCPs from their respective catchment areas. The training process was conducted on the basis of the revised manual of the pilot phase, and involved two steps: a theoretical session during three days, followed by two weeks of practical internship supervised by the head nurse in the health facility serving the HCP’s village. This health facility provided supervision and a link between the HCP and the formal health system.

#### Drugs and supplies

SD Bioline Malaria Ag Pf RDT, an RDT meeting performance criteria for WHO procurement recommendations [21], and having demonstrated good accuracy in the field in Senegal [19] was used to test patients with suspected malaria. Patients with a positive RDT were treated with an artesunate-amodiaquine fixed dose combination that had an efficacy and safety profile confirmed by a local research institution [22]*.* As recommended by the national policy during the period of this intervention, RDTs were performed free of charge. ACT treatments cost USD 0.6 for adults and USD 0.3 for children through April 2010 and since May 2010 have been free of charge for all ages.

A supply and monitoring system was established and drugs and supplies were provided by the supporting health facility. Programme monitoring included post-training follow up, monthly supervision by the health post head nurse, and coordinating meetings carried out by DHTs and the nurses in the peripheral health facilities. Also, at regional level, biannual coordinating meetings were held, involving all stakeholders in the region.

#### Incentives

While there were no monetary incentives and the HCP worked as a volunteer, incentives included recognition by receiving a certificate, job aids and equipment after training, and an official launch organized by the local, administrative and health authorities. In addition, the HCPs received transport fees during regular meetings, participated in mass campaigns of immunization and net distribution, and received consultation free of charge in health facilities when needed.

#### Participation, advocacy and communication

Local and administrative authorities as well as community leaders were informed and involved, and both governmental and non-governmental organizations played a crucial role during the implementation. At the district level, networks of local grassroots organizations that assist DHTs in the implementation of community based-interventions participated in training sessions and meetings as well as advocacy and communication activities. Sensitization meetings were organised by health workers and community leaders in the selected villages, and the implementation was supported by community mobilization events and broadcasts on local radio stations. Lastly, the head of the village provided support to the HCP and ensured the link between the community and the HCP.

### Evaluation

This evaluation was based on data collected both at community level by the HCPs and at health facilities. Most HCPs started reporting data in August 2009, and data were available only through May 2010, effectively limiting the analysis to the first few months of implementation (malaria transmission season of 2009).

At health facilities (hospitals, health centres and health posts), routine malaria data are collected by health workers using a standard form. They report on a monthly basis to the district, where all data are validated and aggregated before entry to the database (Epi Info Version 6) and sent to the NMCP. The NMCP has implemented a data quality assurance system at three stages: first, all reports are validated by the upper level before being submitted, second, a data quality assessment is part of the supervision checklist on all levels including HCPs, and lastly, on a quarterly basis the NMCP holds review meetings with regional and DHTs and all district data (from health facilities and community level) are presented and validated. Finally, the aggregated data for each district are entered into the national database.

The HCPs recorded on standard registers patient demographics, test results, treatment and follow up as well as drug consumption and stock. The information on this register was aggregated by the head nurse at the health post, who prepared a monthly report for the DHT. All of these reports were presented and validated during the coordinating meetings. These data were not integrated into the data from health facilities for the purposes of this analysis.

Data reported by the HCPs were used to assess performance in terms of diagnosis, treatment and referral in 2009 and 2010. Given the simultaneous scale-up of other malaria control interventions including insecticide-treated nets during the same period, it was necessary to identify comparison regions similar in terms of malaria transmission to evaluate impact at the health facility level. The step-wise introduction of HMM, in the majority of targeted regions in 2009, and in the remaining two high transmission regions (Kolda and Sedhiou) in 2010 allowed for comparison of health facility data between the regions in which HMM was introduced in 2009, and the regions in which it was delayed until the following year. Unfortunately, as the scale up of the programme started in August 2009, the time frame available for comparison is limited to the initial months of scale-up.

Other than HMM, the same malaria control strategies were implemented both in intervention and control areas. ITN ownership and use in intervention and comparison regions are shown in Table 1, and were slightly higher in the comparison regions. Routine data reported from the health facilities were used to assess the impact of the intervention, by comparing morbidity and mortality reported during 2009 to that reported in 2008, the baseline year. The NMCP database was used to calculate the numbers of total consultations, suspected malaria cases, confirmed malaria cases, hospitalizations and deaths attributed to malaria for all districts in the intervention and comparison areas. Absolute counts were converted to incidence per 100,000 using district population. Poisson confidence intervals were calculated for the incidence risk difference between 2008 and 2009 in the intervention and comparison regions. These were then used to calculate confidence intervals for the per cent change from 2008 to 2009.

**Table 1:** Household possession and use of insecticide-treated nets in intervention and comparison regions in December 2009*

	**Proportion of households possessing at least one ITN**	**Proportion of children < 5 years sleeping under an ITN**	**Proportion of the total population sleeping under an ITN**
	**% (95% CI)**	**% (95% CI)**	**% (95% CI)**
Intervention regions			
Fatick	96.7 (94.9-98.4)	60.2 (40.2-80.1)	52.2 (37.4 – 67.1)
Kaffrine	89.9 (84.3-95.5)	46.5 (23.3-69.7)	37.5 (17.0-58.1)
Kaolack	95.4 (93.0-97.9)	44.5 (32.4-56.7)	33.8 (23.5-44.0)
Kédougou	87.0 (82.4-91.6)	41.1 (27.9-54.3)	30.4 (22.9-37.9)
Matam	84.0 (77.8-90.3)	58.6 (46.1-71.2)	45.8 (36.7-54.9)
Tambacounda	86.6 (82.3-91.0)	37.1 (26.8-47.5)	26.1 (19.5 – 32.7)
Comparison regions			
Kolda	89.2 (84.3-94.1)	70.8 (63.0-78.5)	60.6 (56.1-65.1)
Sédhiou	90.2 (86.1-94.3)	66.2 (53.2-79.2)	53.0 (42.4-63.7)

*Regional level data from post-ITN distribution campaign survey in December 2009 [23].

Costs for the HMM programme were calculated, including costs for training, non-consumable supplies, and supervision. RDTs and ACTs were supplied by the nearest health post and were not included in the cost calculation, on the assumption that these costs would have been borne by the system in the absence of the programme.

### Ethical considerations

This intervention was driven by the Ministry of Health according to its policy and malaria strategy. Information was given to all communities and local leaders prior to the implementation. Individual patient information was not collected as part of the assessment. As a programme implementation, Institutional Review Board clearance was not required.

## Results

### HCP performance

During 2009, the 408 HCPs saw 6997 patients with suspected malaria, and performed RDTs in 6198 (92.5%). Of those, 2144 (34.6%) were positive, and of those with a positive RDT, 2061 (96.1%) were reported as treated and cured. Most of the HCPs started reporting data after July 2009, at the beginning of the malaria transmission season, and the numbers of cases monthly in 2009 are shown in Figure 2 to demonstrate the seasonality. From January to May 2010, the 861 HCPs saw 5885 cases of suspected malaria, and tested 5474 (93.0%). Of those, 2126 (38.8%) had a positive RDT, and of those with a positive RDT, 97.1% were reported treated and cured. No deaths were attributed to malaria in communities with HCPs (Table 2). In total, 92.8% (11,672/12,582) of patients received an RDT, 36.6% (4270/11672) had a positive RDT, and 96.6% of those (4126/4270) were reported treated and cured. Home-based care providers referred 3377 patients in 2009 and 3494 patients in 2010, primarily for a negative RDT indicating need for further evaluation. Of the 4054 patients with a negative RDT in 2009, 3224 (79.5%) were referred, and in 2010, 3262/3348 (97.4%) of patients with a negative RDT were referred (Table 3).

**Table 2:** Patients and case management by home-based care providers

	**Number of suspected cases seen by HCP**	**Number of RDTs performed**	**Number of positive cases**	**Number of patients treated and cured**	**Number of deaths attributed to malaria in communities with HCPs**
2009	6697	6198	2144	2061	0
2010*	5885	5474	2126	2065	0
TOTAL	12582	11672	4270	4126	0

*Results for 2010 only through May 2010.

**Table 3:** Reasons for patient referral by home-based care provider to the health post

	**Negative RDT**	**Infant < 2 months**	**Pregnant**	**Severe disease**	**TOTAL**
2009	3224	41	36	76	3377
2010	3262	78	69	85	3494
TOTAL	6486	119	105	161	6871

*Results for 2010 only through May 2010.

### Malaria morbidity and mortality

The reported incidence and deaths per 100,000 population were similar in intervention and comparison areas in 2008. The numbers of total outpatient consultations increased in both intervention and control regions, by 15.8% (95% CI 15.5-16.1) and 10.9% (95% CI 10.4-11.4), respectively. Suspected malaria cases per 100,000 decreased by 26.2% (95% CI 25.6-26.8) in 2009 compared to 2008 in intervention regions, and by 10.5% (95% CI 9.5-11.5) in 2009 compared to 2008 in comparison regions, while confirmed cases decreased 27.2% in both. Total and malaria-related hospitalizations decreased similarly in intervention and comparison regions, with total hospitalizations decreasing by 23.6% (95% CI 21.6-25.6) and 24.7% (95% CI 21.4-28.0), respectively, and malaria-related hospitalizations decreasing by 43.1% (95% CI 39.6-46.6) and 40.9% (95% CI 34.6-47.3), respectively. In the intervention regions, total deaths per 100,000 decreased 15.4% (95% CI 5.4-25.4) and deaths attributed to malaria per 100,000 decreased 62.5% (95% CI 43.8-81.2); whereas no statistically significant change in total deaths or malaria related deaths was seen in comparison regions (Table 4).

**Table 4:** Malaria morbidity and mortality reported by health facilities in intervention and comparison regions, at baseline and during implementation

	**Total out-patients**	**Suspected malaria cases**	**Confirmed cases**	**Total hospitalizations**	**Hospitalizations attributed to malaria**	**Total deaths**	**Deaths attributed to malaria**
**Intervention**	(population in 2009: 3,202,760)						
Incidence per 100,000 (2008)	33165	6648	2616	601	181	26	5.6
Incidence per 100,000 (2009)	38401	4906	1905	459	103	22	2.1
Incidence difference (LCL, UCL)	5236 (5137,5335)	−1743 (−1783, -1703)	−711 (−736, -686)	−142 (−154, -130)	−78 (−84, -72)	−4 (−6.6, -1.4)	−3.5 (−4.5, -2.5)
Percent change % (LCL,UCL)	15.8% (15.5, 16.1)	−26.2% (−26.8, -25.6)	−27.2% (−28.1, -26.2)	−23.6% (−25.6, -21.6)	−43.1% (−46.6, -39.6)	−15.4% (−25.4, -5.4)	−62.5% (−81.2, -43.8)
**Comparison**	(population in 2009: 1,021,296)						
Per 100,000 (2008)	32227	7428	2339	611	149	31	4.7
Per 100,000 (2009)	35728	6648	1704	460	87	30	3.6
Incidence difference (LCL, UCL)	3501 (3340, 3662)	−780 (−853, -707)	−636 (−675, -597)	−151 (−171, -131)	−61 (−70, -52)	0 (−4.8, 4.8)	−1.1 (−2.9, 0.7)
Percent change % (LCL, UCL)	10.9% (10.4, 11.4)	−10.5% (−11.5, -9.5)	−27.2% (−28.9, -25.5)	−24.7% (−28.0, -21.4)	−40.9% (−47.3, -34.6)	−1.0% (−15.5, 15.5)	−23.4% (−61.4, 14.5)

### Costs

The scale-up of the programme during 2009 cost a total of USD 163,424.61, excluding RDTs and ACT. Training and supervision for the 408 HCPs cost USD 121,424.61, while non-consumables cost USD 42,000.00. When divided by the number of HCP, cost per community covered was USD 400.55. With an average community size of approximately 500, this amounts to less than USD 1.00 per at risk person.

## Discussion

The scale-up of HMM maintained the high adherence of HCPs to case management guidelines and the absence of malaria-attributed deaths seen in the pilot phase. HCPs tested 93% of suspected cases in both 2009 and 2010, and treated more than 95% of confirmed cases in both years, with no deaths attributed to malaria, showing that the rapid scale-up did not compromise performance. A high proportion of patients with negative RDTs were reported to have been referred for further evaluation.

The increase in overall consultations from 2008 to 2009, with the simultaneous decreases in suspected and confirmed cases as well as malaria-attributed hospitalizations in both areas, is likely due to increased access to health care in the setting of simultaneous scale-up of malaria prevention, primarily long-lasting insecticide treated nets (LLINs), which took place during the same period. A nationwide LLIN distribution campaign targeting children 6–59 months was conducted in June 2009 [23]*.*

The intervention regions experienced a statistically significant decrease in all deaths and deaths attributed to malaria; no such decrease was seen in comparison regions. No other known differences in malaria management were present between comparison and intervention regions (diagnostics, treatment, supportive interventions, and costs). This pattern is consistent with an intervention such as improved access to case management that, while not having an impact on number of cases in the short term, prevents progression to severe disease and death. While there was not a relative decrease in hospitalizations, the HCPs also facilitated timely referral of severe cases, which may have enabled those patients to receive inpatient care while in relatively good condition. While not proof of a causal relationship, it is plausible that a large proportion of deaths are due to severe disease in those who have difficult access to health care and thus have long delays in seeking care, and that home-based diagnosis and treatment for these remote populations prevents the progression of uncomplicated malaria to severe disease and death.

While costs were not formally measured, at a cost of less than $1 per person at risk, programme cost was reasonable. A formal cost-effectiveness analysis of HMM has found it a cost-effective strategy, even when not taking into account the decreased expenses for the users [24]. The cost-effectiveness of HMM would be increased by expanding the services provided by HCPs to include management of diarrhoea and pneumonia, further decreasing the numbers of patients requiring health facility referral. Other roles for home-based care providers have been explored, such as combining home-based management with intermittent preventive treatment in children [25-28].

This analysis faces a number of limitations. In a research setting, one would pre-define and possibly randomize intervention and control areas, and record a great deal more information. In a large-scale implementation in the majority of the regions of the country, this is not feasible, thus analysis of a limited set of programmatic data is necessary. Proving causality is not possible, but sources are triangulated to arrive at an argument for the plausibility of the impact. Data reported by the HCPs are subject to bias as they may inflate their performance, although all data reported by the HCPs were required to be verified by the health post nurse. Data reported by health facilities does not capture those not treated by the public health sector. Only deaths that occurred in public health facilities are measured; deaths occurring at private health facilities, at home or prior to arrival cannot be measured by this method. While a shift of deaths away from public health facilities in the intervention regions is possible, the increase in outpatient consultations in both regions, as well as the identical decrease in hospitalizations in the intervention and comparison regions argues against this. Analysis of impact is limited to the malaria transmission season of 2009, when the intervention was being scaled-up, which likely decreases impact measured. In addition, the implementation was limited to a subset of the most remote communities, and did not cover the entire population for which district level data was reported, possibly further diluting the impact. Given the simultaneous scale-up of other malaria control interventions, the amount of impact is impossible to measure due to the inability to firmly attribute decreases to the HMM programme. Nonetheless, it appears that the implementation of HMM is likely to have been at least partially responsible for the decrease in deaths attributed to malaria in the regions in which it was implemented.

## Conclusion

Rapid scale-up of home-based management of malaria in Senegal demonstrated continued excellent adherence of home care providers in appropriate management of febrile patients based on diagnosis with RDTs and treatment with ACT. The goal of the HMM programme is to continue the scale up to reach 2000 remote communities. In the interim, ACT has been offered free of charge in the public sector since May 2010, and plans have been made to train HCPs to manage diarrhoea and pneumonia. As an increasing proportion of febrile cases are managed in the community, provision of diagnosis-based case management at the community level is imperative in order to measure achievements in malaria control and provide adequate disease surveillance.

## Competing interests

The authors declare that they have no competing interests.

## Authors’ contributions

ST coordinated this study. ST and JT have designed the methodology, done the analysis and wrote the manuscript. ID and FBF carried out the planning, training, implementation and monitoring during the study. MN assisted with data collection, data cleaning and analysis. MBD and RP were involved in the study implementation and analysis. MLD and MC participated in the training and monitoring. MMD assisted with planning, training and supervision. MT wrote the protocol with ST, oversaw this intervention and revised the manuscript. All authors read and approved the manuscript.
